# Identifying clinical subgroups in IgG4-related disease patients using cluster analysis and IgG4-RD composite score

**DOI:** 10.1186/s13075-019-2090-9

**Published:** 2020-01-10

**Authors:** Jieqiong Li, Yu Peng, Yuelun Zhang, Panpan Zhang, Zheng Liu, Hui Lu, Linyi Peng, Liang Zhu, Huadan Xue, Yan Zhao, Xiaofeng Zeng, Yunyun Fei, Wen Zhang

**Affiliations:** 1 Department of Rheumatology, Peking Union Medical College Hospital, Chinese Academy of Medical Sciences & Peking Union Medical College, National Clinical Research Center for Dermatologic and Immunologic Diseases (NCRC-DID), Key Laboratory of Ministry of Health, Beijing, China; 2 Central Research Laboratory, Peking Union Medical College Hospital, Beijing, China; 3 Department of Radiology, Peking Union Medical College Hospital, Beijing, China

**Keywords:** IgG4-related disease, Laboratory test, Organs involved, Cluster analysis, IgG4-RD CS

## Abstract

**Background:**

To explore the clinical patterns of patients with IgG4-related disease (IgG4-RD) based on laboratory tests and the number of organs involved.

**Methods:**

Twenty-two baseline variables were obtained from 154 patients with IgG4-RD. Based on principal component analysis (PCA), patients with IgG4-RD were classified into different subgroups using cluster analysis. Additionally, IgG4-RD composite score (IgG4-RD CS) as a comprehensive score was calculated for each patient by principal component evaluation. Multiple linear regression was used to establish the “IgG4-RD CS” prediction model for the comprehensive assessment of IgG4-RD. To evaluate the value of the IgG4-RD CS in the assessment of disease severity, patients in different IgG4-RD CS groups and in different IgG4-RD responder index (RI) groups were compared.

**Results:**

PCA indicated that the 22 baseline variables of IgG4-RD patients mainly consisted of inflammation, high serum IgG4, multi-organ involvement, and allergy-related phenotypes. Cluster analysis classified patients into three groups: cluster 1, inflammation and immunoglobulin-dominant group; cluster 2, internal organs-dominant group; and cluster 3, inflammation and immunoglobulin-low with superficial organs-dominant group. Moreover, there were significant differences in serum and clinical characteristics among subgroups based on the CS and RI scores. IgG4-RD CS had a similar ability to assess disease severity as RI. The “IgG4-RD CS” prediction model was established using four independent variables including lymphocyte count, eosinophil count, IgG levels, and the total number of involved organs.

**Conclusion:**

Our study indicated that newly diagnosed IgG4-RD patients could be divided into three subgroups. We also showed that the IgG4-RD CS had the potential to be complementary to the RI score, which can help assess disease severity.

## Background

IgG4-related disease (IgG4-RD) is a multi-organ immune-mediated condition characterized by tumefactive lesions, a dense lymphoplasmacytic infiltrate rich in IgG4-positive plasma cells, storiform fibrosis, and, often but not always, elevated serum IgG4 concentrations [1–3]. The comprehensive diagnostic criteria for IgG4-RD were published in 2011 by the Umehara and Okazaki teams [4]. The IgG4-RD responder index (RI) is a disease activity tool modeled by Stone et al. [5, 6], wherein the sum of organ sites’ score (each organ/site graded on a 0–4 scale) plus the serum IgG4 concentration score (0–4) yields the total RI score. In 2015, an international consensus guidance statement on the management and the treatment of IgG4-RD was published [7]. Glucocorticoids (GCs) are the first-line agents for remission induction; some patients require the combination of GCs and steroid-sparing immunosuppressive agents (IMs) [7–12]. B cell depletion with rituximab (RTX) is also effective at inducing remission in IgG4-RD [13–15].

An increasing number of studies on the outcomes and prediction of disease relapse in IgG4-RD showed that high baseline serum IgG4, IgE, and eosinophilia could predict IgG4-RD relapse independently [15]; eosinophilia, higher baseline RI, having five or more organs involved, and dacryoadenitis were risk factors for remission induction failure with GC monotherapy [16]; and hypocomplementemia was more frequent in IgG4-related kidney disease (IgG4-RKD), which may participate in the disease development [17, 18]. However, the disease subclass, outcomes, and predictors of disease relapse in IgG4-RD still require further exploration.

To our knowledge, no classification of IgG4-RD based on diversified routine blood tests has been proposed. In this study, we aimed to explore the baseline clinical patterns of IgG4-RD based on 19 different types of blood tests and the number of organs involved. We also tried to establish a prediction model for a comprehensive score (IgG4-RD composite score, IgG4-RD CS), which was expected to assess disease severity and partly predict outcomes in IgG4-RD patients.

## Methods

### Study population

This study was approved by the Ethics Committee of Peking Union Medical College Hospital (PUMCH). All the patients provided written and informed consent. The prospective cohort of patients with IgG4-RD in the PUMCH [19] was registered on ClinicalTrials.gov (NCT01670695). All the patients fulfilled the 2011 comprehensive diagnostic criteria for definite, probable, or possible IgG4-RD [4]. Patients were recruited from 2014 to 2018. The criteria for IgG4-RD patient inclusion in this study were as follows: (1) newly diagnosed patients without treatment and (2) no missing data in 19 different types of blood tests. Data pertaining to demographics, treatments, and laboratory findings at baseline were derived from the medical records.

### Baseline clinical evaluation and laboratory assessments

Age at onset refers to the age at which the patient first noticed the symptoms attributed to IgG4-RD or to the timepoint at which the disease was first recognized (whichever was earlier) [3]. Disease duration refers to the period of time from first symptoms attributed to IgG4-RD to initial diagnosis. Allergic disease history was recorded and confirmed by enquiring each patient, previous allergen detection, and diagnostic reports. The number of organs involved was determined by a review of the patient’s history, physical examination, blood tests, imaging, and tissue biopsies. The organs involved were divided into superficial organs (i.e., salivary glands, lacrimal glands, orbit, sinus, and skin) and internal organs (i.e., all of the other organs) [20–22]. Lymph nodes were not taken into account when assessing the involvement of the superficial/internal organs [20]. Disease activity was assessed using the 2012 IgG4-RD RI [5], and the involved organs were evaluated individually. Patients were defined into different RI score grades by separating the RI range of 154 patients into trisection by average.

All obtainable baseline data was recorded before initial treatment. Laboratory tests consisted of complete blood count, urinalysis, liver, and renal function tests, erythrocyte sedimentation rate (ESR), hyper-sensitivity C-reactive protein (hsCRP), complement 3 (C3), C4, serum immunoglobulin (Ig) levels, and total IgE and IgG subclasses. To facilitate application, 19 variables from the laboratory tests involved in this study were the most common routine tests in IgG4-RD clinical practice, including white blood count, lymphocyte count, lymphocyte percentage, eosinophil count, eosinophil percentage, platelet count (Plt), hemoglobin (Hb), ESR, hsCRP, C3, C4, IgG, IgA, IgM, IgE, IgG1, IgG2, IgG3, and IgG4.

### Definition of disease response

Disease response after initial treatment was assessed after 3 months. According to the IgG4-RD RI, disease response was defined as an improvement of the IgG4-RD RI ≥ 2 compared to the baseline [14], and compared to IgG4-RD RI < 3 as complete response (CR), IgG4-RD RI remained ≥ 3 as partial response (PR). No change (NC) was defined as an absence of marked changes in mass sizes, organomegaly and/or symptoms and change of IgG4-RD RI < 2 points [8, 9, 23].

### Goals of remission induction

The initial 6-month period was defined as the remission induction stage, and the therapeutic goals of this stage were defined as fulfilling each of the following: (1) ≥ 50% decline in the IgG4-RD RI, (2) GC tapered to maintenance dose (prednisone ≤ 10 mg/day), and (3) no relapse during GC tapering (within 6 months) [16].

### Definition of relapse

Relapse was defined by a review of the new progress or recurrence of symptoms and signs and the new development or return of abnormal findings on physical examination, laboratory tests that reflected IgG4-RD activity within specific organs, or imaging studies [8, 9, 15, 16]. The date of relapse was either the date of symptom onset or new or worse physical examination, laboratory, or radiology findings. An isolated increase in the serum IgG4 concentration did not constitute a disease relapse [15].

### Statistical analysis

All statistical tests were performed using IBM SPSS (version 24). The continuous normally distributed data is presented as mean ± standard deviation, non-normally distributed data is presented as median (first quartile, third quartile), and categorical data is presented as a percentage (%). Comparisons of continuous data such as levels of IgG4 among 3 groups were tested using the one-way ANOVA or the Kruskal-Wallis test. Multiple comparisons were performed using the Student-Newman-Keuls test. Categorical variables were assessed using the chi-square or Fisher’s exact tests. We also used the chi-square tests to compare the proportion of patients with disease relapse among the different subgroups. We calculated the correlation coefficients among the blood test results such as correlations between IgE and eosinophil count using the Pearson correlation coefficient (normally distributed) or the Spearman correlation coefficient (nonparametric data). A two-sided *P* value < 0.05 was considered significant for all statistical testing.

For easy exploration and visualization of 22 baseline variables, which contained 19 variables from laboratory tests and the number of total, internal, and superficial organs involved, and for estimation of the correlation between variables, we used PCA to statistically aggregate these variables, reducing the number of observed variables into a smaller number of principal components (PCs) and reducing the dimensionality of baseline phenotyping data. According to eigenvalues and cumulative contribution rate, we selected the appropriate number of PCs (PC1, PC2, …, PCi) with high eigenvalues (*λ* > 1). According to Component Matrix and eigenvalue, PC scores (the values for extracted PCs) were also calculated in each patient as new variables (F1, F2, …, Fi) for further cluster analysis. Cluster analysis was performed using a hierarchical and agglomerative clustering algorithm with the Ward method [24, 25], using the aforementioned new variables of each IgG4-RD patient. We determined the number of clusters based on the tree diagram.

Calculation of IgG4-RD CS involved two steps. First, PCA was applied to 22 variables to derive PCs representing large fractions of variance. Second, IgG4-RD CS was computed by summing individual PC scores (F1, F2, …, Fi) with the contribution rate(C = *λ*/*p*, *p* = 22) as weight coefficient for each patient (IgG4-RD SC = C1F1 + C2F2 + ... + CiFi), which enabled us to group patients according to their IgG4-RD CS grades by separating the IgG4-RD CS range into trisection averages. Finally, a mathematical model for the “IgG4-RD CS” was established using multivariate linear regression.

**Fig. 1:**
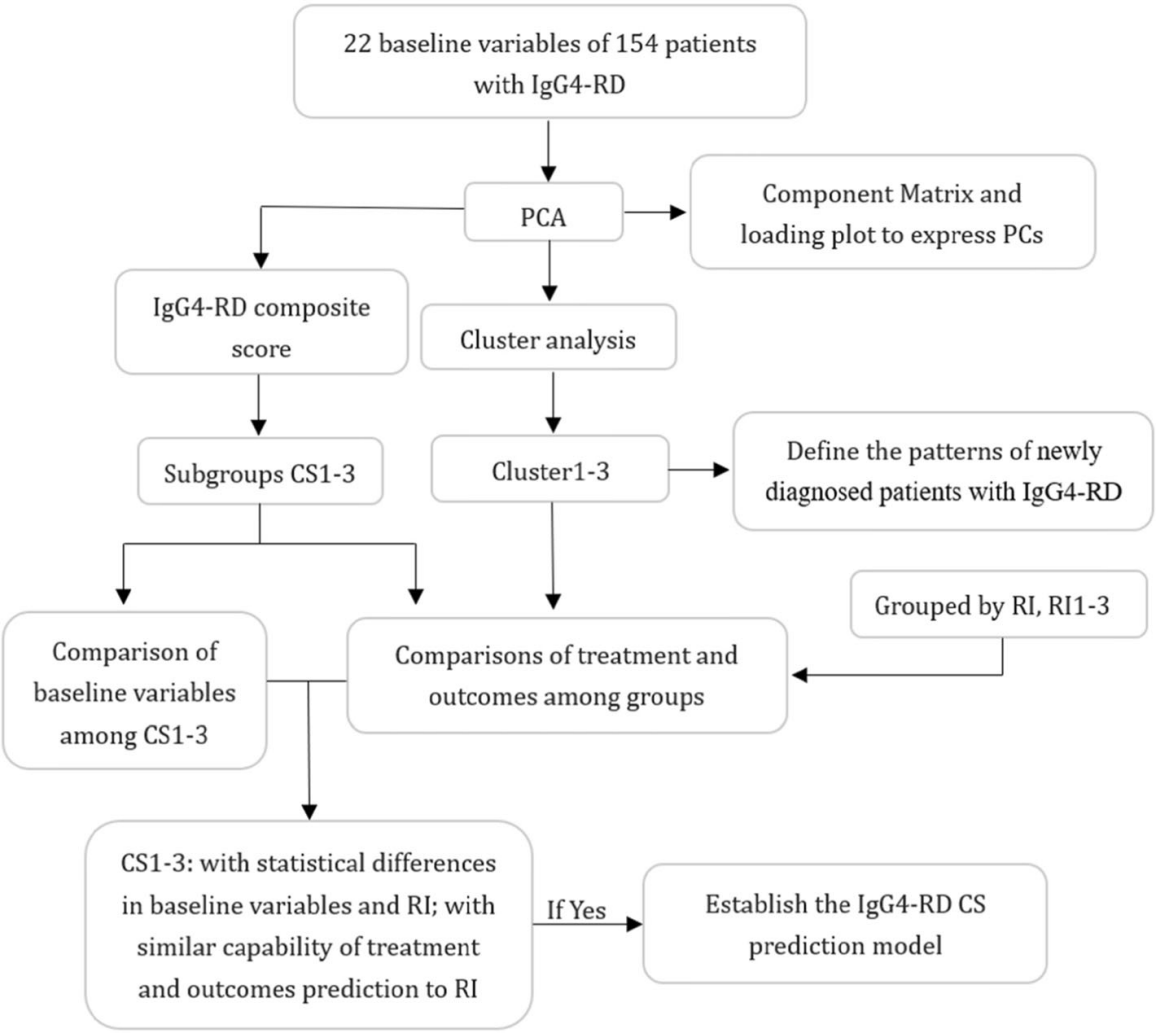
The flow scheme of statistical analysis, patients’ grouping, and comparisons among subgroups. PCA, principal components analysis; PCs, principal components; RI, IgG4-RD responder index (2012); IgG4-RD CS, IgG4-RD composite score

The flow scheme of statistical analysis, patients grouping, and comparisons among subgroups is shown in Fig. 1.

## Results

### Demographic and clinical characteristics

One hundred and fifty-four newly diagnosed patients were included in this study. There were 99 male and 55 female patients (male to female 1.8:1). The average age at onset was 53.35 years (± 13.2, range 9–83). The median duration of disease was 11.5 months (range 10 days to 20 years). There were 68 (44.16%) patients with a history of allergies (male to female 1.5:1). There were 87 (56.49%), 3 (1.95%), and 64 (41.56%) cases, which were diagnosed as definite, probable, and possible IgG4-RD, respectively.

Of the 154 patients, the median number of the total organs involved was 3 (range 1–8). About 30% (46/154) had only superficial organ involvement, and 31% (48/154) had only internal organ involvement. The median IgG4-RD RI was 11.5 (range 3–28). The detailed demographic and clinical characteristics are shown in Additional file 1.

### Correlations of serological variables

With regard to the correlations between serological variables, we mainly focused on IgG4, IgG, IgE, ESR, and CRP. Serum IgG4 concentration showed positive correlations with serum IgG concentration (*r* = 0.604, *P* < 0.001), IgE (*r* = 0.359, *P* < 0.001), and IgG3 (*r* = 0.272, *P* = 0.001) and negative correlations with IgA (*r* = − 0.442, *P* < 0.001), IgM (*r* = − 0.265, *P* = 0.001), C3 (*r* = − 0.338, *P* < 0.001), and C4 (*r* = − 0.41, *P* < 0.001).

Serum IgG concentration correlated positively with IgE (*r* = 0.243, *P* = 0.002), ESR (*r* = 0.587, *P* < 0.001), CRP (*r* = 0.225, *P* = 0.005), as well as Plt (r = 0.204, *P* = 0.011) and negatively with C3 (r = − 0.193, *P* = 0.016), C4 (*r* = − 0.315, *P* < 0.001), and Hb (*r* = − 0.36, *P* < 0.001).

As for serum IgE levels, positive correlations were found between IgE and eosinophil count (*r* = 0.257, *P* = 0.001), as well as eosinophil proportion (*r* = 0.25, *P* = 0.002).

We also investigated the correlations between RI and serological variables. RI scores showed positive correlations with serum IgG4 (*r* = 0.589, *P* = < 0.001), IgG (*r* = 0.333, *P* < 0.001), IgE (*r* = 0.267, *P* = 0.001), IgG3 (*r* = 0.231, *P* = 0.004), and eosinophil count (*r* = 0.223, *P* = 0.005) as well as eosinophil proportion (*r* = 0.229, *P* = 0.004) and negative correlations with IgA (*r* = − 0.366, *P* < 0.001), C3 (*r* = − 0.389, *P* < 0.001), and C4 (*r* = − 0.488, *P* < 0.001).

### Clinical phenotypes based on PCA

**Fig. 2:**
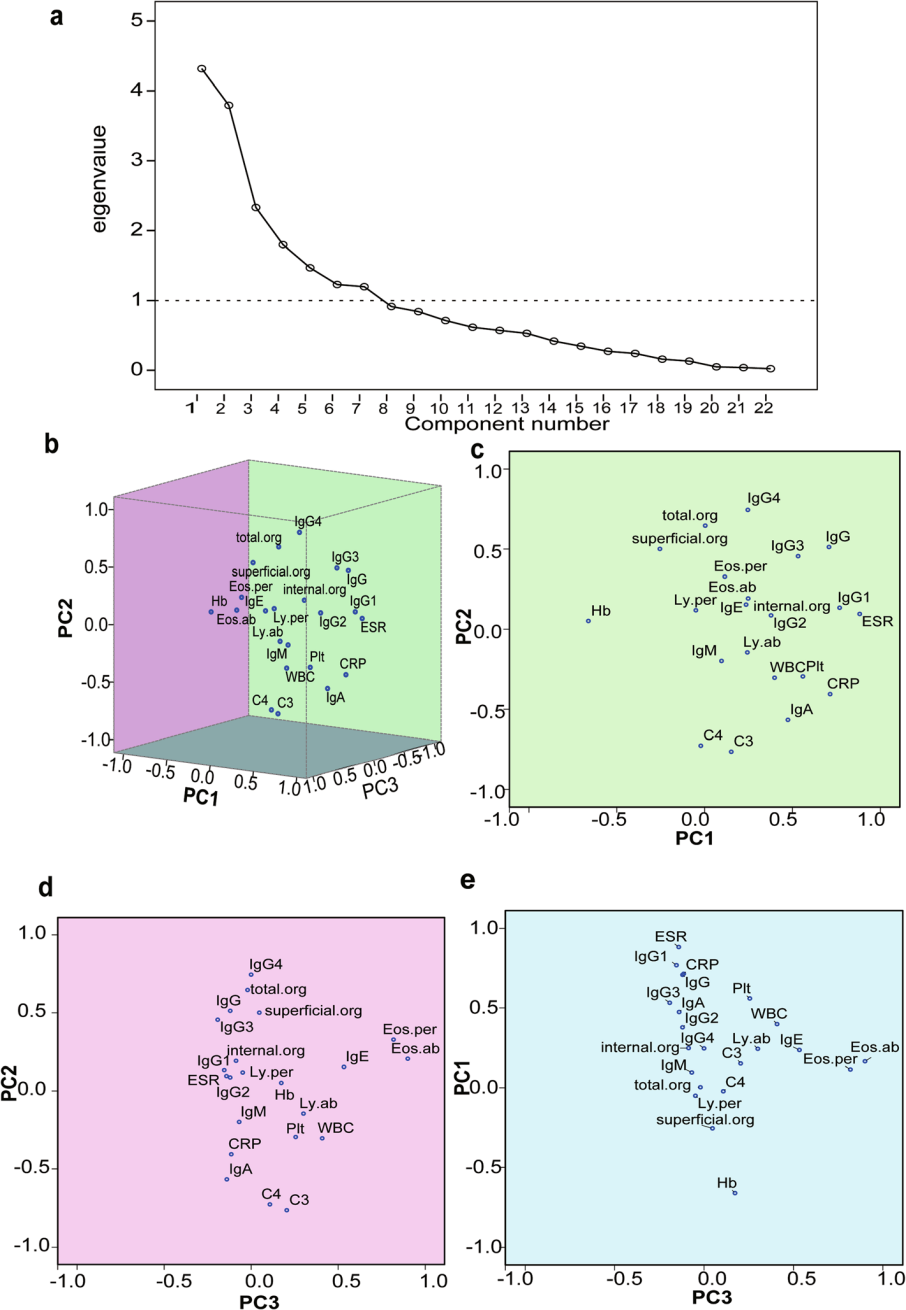
Results of PCA based on 22 baseline variables. **a** Scree plot. Every hollow spot represents one principal component. Vertical axis shows eigenvalue of each spot. **b**–**e** Loading plot. Each baseline variable was visualized in 3 dimensions (**b**). Planes consisting of axis PC1 and PC2, PC2 and PC3, and PC3 and PC1 are colored green, light purple, and blue. LY.ab, absolute lymphocyte count; Ly.per, lymphocyte percentage; Eos.ab, absolute eosinophil count; Eos.per, Eosinophil percentage; total.org, the number of total organs involved; superficial.org, the number of superficial organs involved; internal.org, the number of internal organs involved

Seven PCs (eigenvalue, *λ*1–7 = 4.317, 3.794, 2.33, 1.798, 1.468, 1.23, 1.198) were extracted from 22 baseline variables by PCA, and the cumulative score was up to 73.34% (Fig. 2a and Additional file 2). The correlation coefficient shown in the Component Matrix reflected the ability of each PC to represent the original variable, and baseline variables were statistically aggregated to the 7 PCs (Table 1). For easier exploration and visualization of these baseline variables, we also created a 3-dimensional loading plot as well as 2-dimensional images from different plane angles (Fig. 2b–e). The PCA results showed that PC1 was associated with the inflammatory phenotype. The positive side of PC1 contained ESR, CRP, IgG1, IgG3, IgG, and Plt, while the negative side contained Hb. PC2 seemed to be mainly associated with the disease severity of IgG4-RD, for the positive side contained IgG4 and number of the total organs involved. PC3 predominantly showed allergy-related variables, as the positive side contained IgE, eosinophil count, and eosinophil proportion. The correlation coefficient was also shown in the Component Matrix of the first 3 PCs (Additional file 3). Additionally, we further explored the distances between variables to help clarify the correlations among the baseline variables (Fig. 2b–e). Both C3 and C4 were statistically opposed to and showed negative correlations with the number of total organs involved as well as that of superficial organs involved (*P* < 0.001). IgA and CRP were statistically close and showed positive correlation (*r* = 0.214, *P* = 0.008). The results of the PCA indicated that the baseline phenotypes of IgG4-RD basically consisted of the inflammation-related axis, disease severity-related axis, and allergy-related axis abnormalities.

**Table 1:**
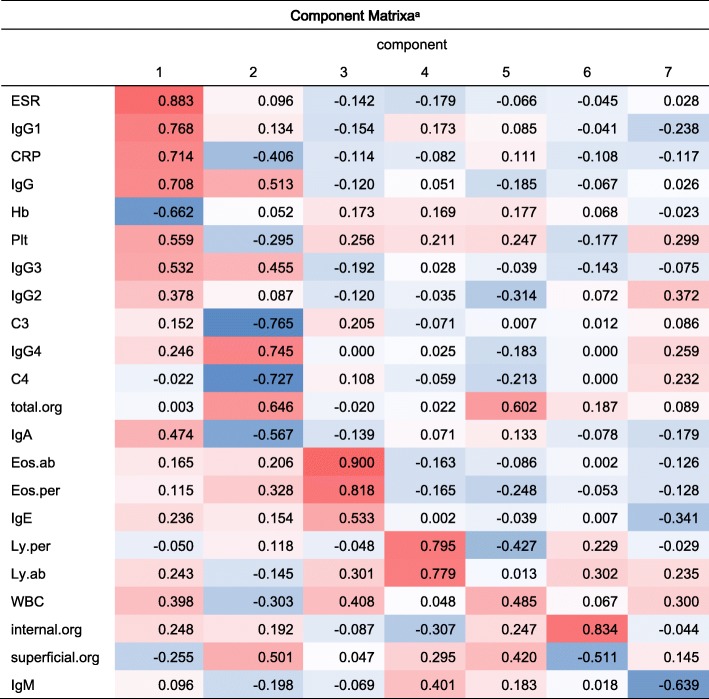
Correlation coefficient of each phenotype after extracting 7 PCs

^a^Seven components were extracted. Red represents the positive value, blue represents the negative value. The color depth of each cell is proportional to the absolute value of correlation coefficient

### Clinical subgroups by cluster analysis

**Fig. 3:**
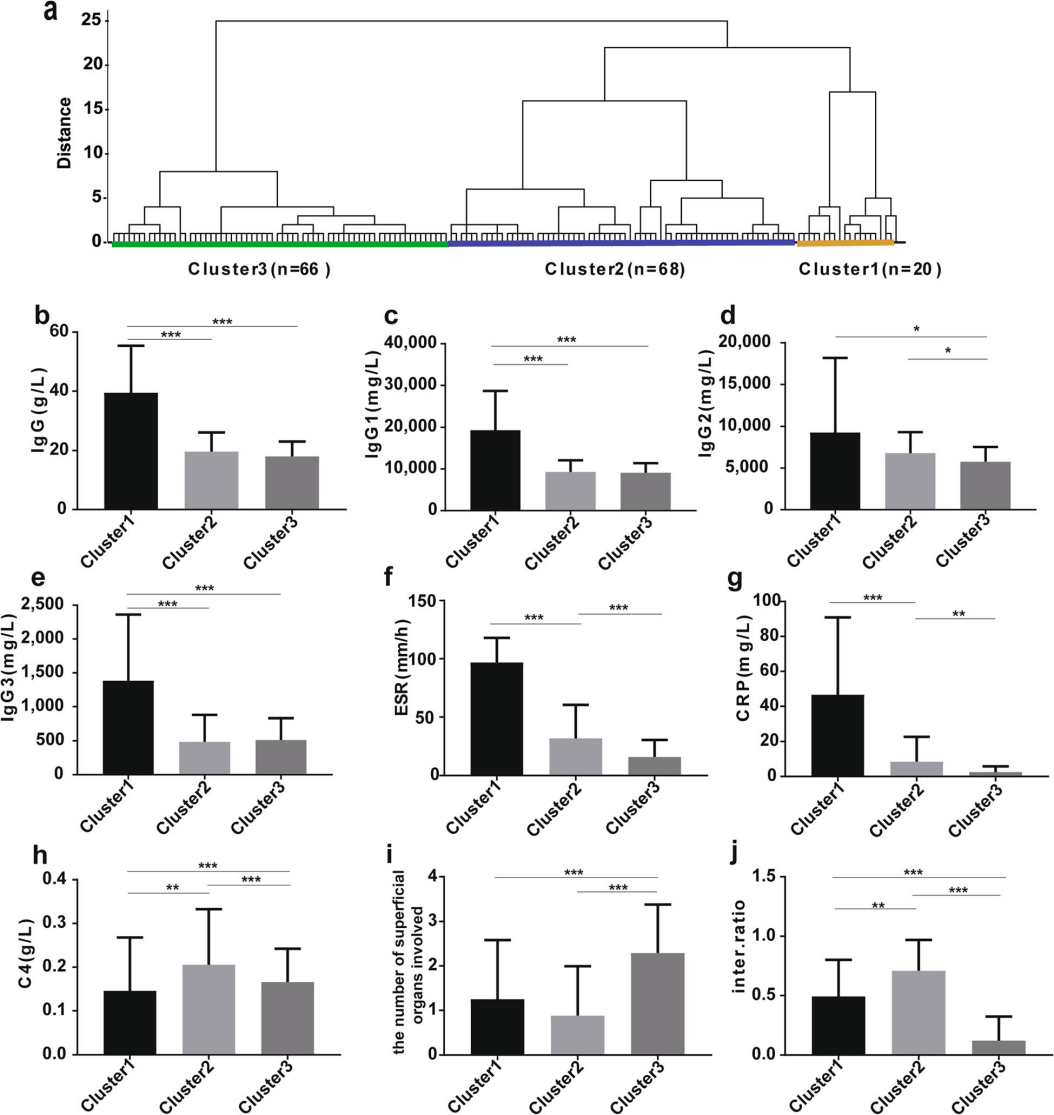
Results of cluster analysis and differences among clusters. **a** Three clusters of patients identified by cluster analysis. Hierarchical statistical clustering of IgG4-RD patients. **b**–**j** Comparisons of baseline characteristics among three clusters of IgG4-RD patients. Inter.ratio, proportion of internal organs to total organs involved. *P value < 0.05; **P value < 0.01; ***P value < 0.001

Based on the 7 PCs, cluster analysis revealed that the 154 newly diagnosed patients with IgG4-RD could be classified into 3 subgroups (termed clusters 1, 2, 3; *n* = 20, *n* = 68, *n* = 66, respectively) (Fig. 3a). We explored the baseline features of the 3 clusters (Table 2). The major statistical differences among clusters also are shown in Fig. 3. Cluster 1 had the highest serum levels of IgG, IgG1, IgG3, ESR, and CRP, but the lowest level of C4 (termed the inflammation and immunoglobulin-dominant group). Cluster 2 had the highest proportion of internal organ to total organ involvement (termed the internal organs-dominant group). In contrast, cluster 3 had the highest number of superficial organs involved, while the lowest proportion of internal organ to total organ involved, and the lowest levels of ESR, CRP, and IgG2 (termed inflammation and immunoglobulin-low with superficial organ-dominant group).

**Table 2:** Baseline characteristics of patients with IgG4-RD grouped by cluster analysis

	Cluster 1 *n* = 20	Cluster 2 *n* = 68	Cluster 3 *n* = 66	*p* value
Sex (male; female)	2.33:1	3.533:1	0.94:1	0.002
Age (years)	54.3 ± 12.24	55.07 ± 14.66	51.29 ± 11.63	0.237
Disease duration (months)	12 (7, 24)	6 (2, 22.5)	12 (6, 36)	0.667
IgG4-RD RI	12.5 (10, 18.25)	12 (7, 15)	11 (7, 15)	0.203
Allergy history, *n* (%)	5 (25%)	33 (48.53%)	30 (45.45%)	0.17
Number of total organs involved	3 (2, 4.75)	3 (2, 4)	3 (2, 4)	0.3
Number of internal organs	2 (1, 2)	2 (1, 2.75)	0 (0, 1)	< 0.001
Number of superficial organs	1 (0, 2)	0 (0, 2)	2 (1, 3)	< 0.001
Internal organs ratio	0.41 (0.33, 0.67)	0.67 (0.5, 1)	0 (0, 0.25)	< 0.001
Laboratory test at baseline				
WBC (×10^9^/L)	7.93 (5.72, 8.33)	7.03 (5.86, 8.36)	6.09 (5.37, 7.03)	0.002
Eosinophils (×10^9^/L)	0.35 (0.11, 0.69)	0.23 (0.09, 0.45)	0.23 (0.14, 0.37)	0.615
Eosinophils (%)	3.45 (1.43, 7.45)	3.15 (1.25, 5.95)	3.4 (1.98, 6.53)	0.836
Lymphocyte (×10^9^/L)	2.05 (1.78, 2.51)	2.09 (1.55, 2.58)	1.70 (1.54, 2.27)	0.091
Lymphocyte (%)	29.24 (23.30, 33.79)	30.29 (22.16, 36.20)	30.73 (25.34, 36.82)	0.667
Hemoglobin(g/L)	115 (98, 125)	138 (125.25, 149)	140.5 (131, 153.75)	< 0.001
Plt (×10^9^/L)	303 (227.5, 386.75)	242 (180.5, 277.75)	232 (206, 282)	0.001
ESR (mm/h)	97 (80.5, 112.75)	22 (7.25, 41.5)	11 (5, 25)	< 0.001
CRP (mg/L)	44.54 (5.01, 78.37)	2.64 (0.74, 9.61)	1.19 (0.36, 3.73)	< 0.001
IgG (g/L)	39.77 (24.48, 47.84)	18.40 (14.74, 23.43)	17.53 (13.87, 21.40)	< 0.001
IgA (g/L)	2.74 (0.85, 5.02)	2.23 (1.59, 2.84)	1.79 (1.33, 2.34)	0.087
IgM (g/L)	0.88 (0.47, 1.57)	0.87 (0.58, 1.17)	0.90 (0.58, 1.41)	0.48
IgG1 (mg/L)	15,100 (12,125, 29,900)	9175 (7242.5, 11,000)	8750 (7290, 10,200)	< 0.001
IgG2 (mg/L)	7455 (5127.5, 9942.5)	6560 (5060, 8575)	5535 (4415, 6962.5)	0.021
IgG3 (mg/L)	1035 (590.25, 2240)	289.5 (195, 662)	422.5 (263, 711.5)	< 0.001
IgG4 (mg/L)	12,550 (2860, 52,975)	8535 (3085, 14,250)	7895 (4235, 14,725)	0.261
IgE (kU/L)	546.5 (163.25, 1340.5)	275 (151.75, 855.75)	369 (99.2, 632.25)	0.183
C3 (g/L)	0.88 (0.39, 1.51)	1.03 (0.86, 1.19)	0.95 (0.75, 1.04)	0.077
C4 (g/L)	0.14 (0.03, 0.20)	0.20 (0.12, 0.27)	0.17 (0.13, 0.21)	0.03
IgG4-RD CS	1.13 (0.37, 1.31)	− 0.06 (− 0.43, 0.26)	− 0.22 (− 0.48, − 0.08)	< 0.001

The continuous normally distributed data are presented as mean ± standard deviation; non-normally distributed data are presented as median (first quartile, third quartile)

To further clarify the situation of complement in clusters, we divided the levels of C3 and C4 into 3 grades respectively: low, normal, and high-levels (Additional file 4a). We found significant statistical differences in both C3 and C4 among clusters 1–3 using chi-square tests (*P* < 0.001, *P* = 0.043, respectively). Cluster 1 had the highest percentage of patients with low-level complement (45%), while most patients in cluster 2 and cluster 3 had normal-level complement (73.53%, 75.75%).

There was no statistical difference in the RI among the 3 clusters (Table 2). These findings indicated that the baseline features were different even among patients with similar RI. Indeed, it was difficult to distinguish subgroups adequately according to RI only.

In addition, we repeated cluster analysis in different genders. The results showed that IgG4-RD patients also could be classified into three clusters no matter in male or female patients: cluster 1, inflammation and immunoglobulin-dominant group; cluster 2, internal organs-dominant group; cluster 3, superficial organs-dominant group (Additional files 5, 6, and 7).

### Group by IgG4-RD CS

Each patient was given a unique IgG4-RD CS by the aforementioned synthetical constructor (IgG4-RD CS=C1F1 + C2F2 + ... + C7F7) as the comprehensive reflection of 22 baseline variables. All 154 patients had a median CS of − 0.1149 (range − 1.223 to 1.996).

Next, we defined IgG4-RD CS grades by dividing the range into trisection by average (grade 1, range − 1.5 to − 0.3; grade 2, range − 0.3 to 0.9; grade 3, range 0.9 to 2.1) and divided 154 patients into 3 groups (termed CS1, 2, 3; *n* = 51, *n* = 90, *n* = 13) accordingly. Then, we compared 22 baseline variables among CS1–3. As expected, we found statistical differences in RI, the number of internal organs involved, the number of total organs involved, lymphocyte count, eosinophil count, ESR, Hb, IgG, IgG1, IgG3, IgG4, C4, and other variables among CS1–3 (Additional files 9 and 10). These findings indicated that the synthetical constructor was successful and able to represent the baseline features for each patient to a great extent, especially the disease severity.

We also explored the relation of the IgG4-RD CS grades to clusters. Interestingly, there were significant differences in the IgG4-RD CS among the 3 clusters, and cluster 1 had the highest percentage of CS3, while cluster 3 had the highest percentage of CS1 (Additional file 4b-c).

### Treatment and outcomes

Among 154 IgG4-RD patients, treatment regimens mainly included GC monotherapy, GC combined with IMs, and GC-sparing medications or “watchful waiting” (16.9%, 60.8%, 22.3%, respectively). We explored the treatment response, remission, and disease relapse. There were 5 patients with no change to treatment, 51 patients with partial responses, and 64 patients with complete responses. Twelve patients (13.3%) failed to achieve remission among those with GC therapy. There were 31 patients (20.1%) who had experienced relapse or were in recurrent states.

**Fig. 4:**
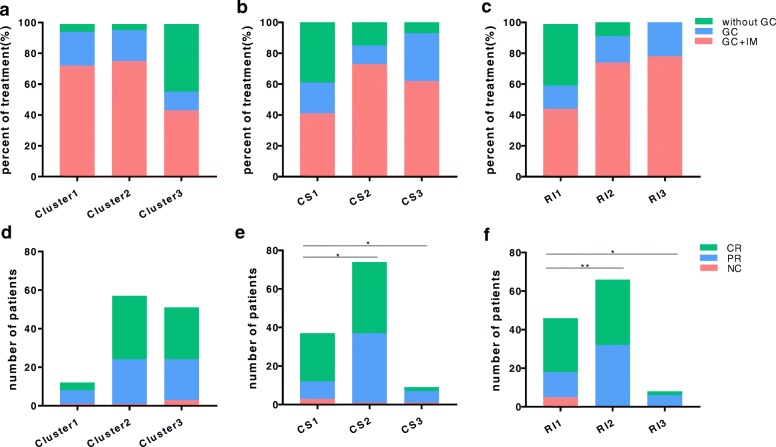
Comparisons of treatments and disease responses among subgroups. **a**–**c** Percent of the treatment regimens among different subgroups. **d**–**f** Disease responses were compared among subgroups. CR, complete response; PR, partial response; NC, no change. *P value < 0.05; **P value < 0.01

We divided 154 patients into 3 new groups (termed RI 1, 2, 3; *n* = 71, *n* = 74, *n* = 9, respectively) again according to the RI score by dividing the range into trisection by average (range 3–28; RI1, range 0–10; RI2, range 11–20; RI3, range 21–30) and explored the differences in the treatment regimens among different subgroups using chi-square tests (Fig. 4a–c). There were significant statistical differences in treatment among clusters 1–3, CS1–3, and RI 1–3 (*P* < 0.001, *P* = 0.002, *P* < 0.001, respectively), and both CS1 and RI1 had the highest proportion of patients without GC treatment (38.8%, 40.0%, respectively). Interestingly, we also found IgG4-RD CS had superior ability to guide treatment in female patients. In male patients, IgG4-RD CS and IgG4-RD RI shared the similar trends corresponding to clusters; cluster 1 and cluster 2 showed higher disease severity scores (both RI and IgG4-RD CS), which was consistent with higher probability of receiving active treatments (GC or GC+IM) (Additional file 8 a, c). In female patients, however, RI was the lowest in cluster 1 but highest in cluster 3, and female patients in cluster 1 received active intervention proportionately with higher IgG4-RD CS but lower RI (Additional file 8 b, d).

In total patients, there were significant statistical differences in disease response among CS1–3 and RI1–3 (*P* = 0.019, *P* = 0.0058, respectively), and subgroup CS1 had the highest proportion of patients with CR as well as subgroup RI1 (Fig. 4d–f). However, no difference was found in remission induction or disease relapse (Additional file 11 a-b).

Considering that the recurrence rate was time-varying, we explored the dynamic changes of relapse situations among groups. There was no difference in the cumulative relapse rate as observed using Kaplan-Meier curves, but we found the half-relapse time was different among groups (Additional file 12 a-c). Cluster 2, CS2, and RI2 had the relatively long half-relapse time than cluster 3, CS1, and RI1, respectively. In addition, we also showed the real-time proportion of non-relapse patients in follow-up patients (Additional file 12d-i), for the patients in subgroup CS3 was not enough to form a curve; the figure of CS3 has not been shown.

### Establishment of IgG4-RD CS prediction model for assessment of IgG4-RD

We used stepwise multiple linear regression to establish an IgG4-RD CS prediction model for IgG4-RD. The multiple regression equation was “IgG4-RD CS = 0.038*IgG(g/L) + 0.385*eosinophil count (10^9^/L) + 0.247*lymphocyte count (10^9^/L) + 0.135*total organs involved number − 1.852”. The 95% confidence interval (95% CI) of these four independent variables and constants were shown as follows: IgG (0.035, 0.04), eosinophil count (0.329, 0.44), lymphocyte count (0.209, 0.285), total number of organs involved (0.118, 0.151), and constant (− 1.958, − 1.746). The model had excellent adjusted *R* square (*R*^2^adj, 0.918) and Durbin-Watson value (DW = 1.842). The plot of residuals also indicated that the model was a statistical success (Additional file 13).

## Discussion

In this paper, we identified abnormalities of the baseline phenotype of IgG4-RD and classified IgG4-RD patients into subgroups based on 22 baseline variables using PCA and cluster analysis. We also calculated the “IgG4-RD CS” as a comprehensive score for each patient using principal component evaluation and explored its capability of disease severity assessment.

IgG4-RD is a heterogeneous disease [26]. Wallace et al. [27] identified four distinctive IgG4-RD phenotypes according to organ involvement using latent class analysis. Regarding the heterogeneity in serology, one of the major characteristics of IgG4-RD is elevation of serum IgG4, and studies have generally attached crucial importance to serum IgG4 concentrations, which is also used to assess disease activity [4, 5, 28–30]. However, serum elevation of IgG4 is the lack of specificity, and normal serum IgG4 concentrations are described even in the setting of active, biopsy-proven disease [3, 31, 32]. Furthermore, a high percentage of eosinophil; high levels of IgG, IgE, ESR, and CRP; and hypocomplementemia have been frequently reported in IgG4-RD and have been related to outcomes [15, 18, 29, 33], but without excellent specificity. Herein, we proposed clinical patterns in IgG4-RD using cluster analysis based on various baseline blood tests.

PCA showed major abnormalities in IgG4-RD (Fig. 2b–e, Table 1, Additional file 3), which have also been described in earlier studies [34, 35]. Complement levels showed negative correlations with IgG4, IgG, and RI and were at the opposite sites of organs involved number in the PCA graph (Fig. 2b–e), which is consistent with the conclusion reported in previous studies that hypocomplementemia is related to disease activity [33]. We also found that IgA had a negative correlation with IgG4. Though there are few studies on the role of IgA in IgG4-RD, a recent study has reported that patients with relapse had significantly higher levels of serum IgG4 but lower levels of serum IgA compared to patients without relapse [30], and the lower level of serum IgA is used to differentiate IgG4-RD from hyper-interleukin (IL-) 6 syndromes such as multicentric Castleman’s disease [36, 37].

In this study, IgG4-RD patients could be stratified statistically into three clusters (Fig. 3a). High levels of inflammation and immunoglobulin were especially noted in cluster 1 (Fig. 3b–j). Moreover, about 33% of patients in cluster 1 failed to achieve remission induction during the 6-month period (Additional file 11a), which was partly because of a small sample size and heterogeneity of follow-up duration, in addition to the disease characteristics of cluster 1. Cluster 2 had a high percentage of internal organ involvement (Fig. 3j). Patients in cluster 2 had the longest half-relapse time of about 48 months (Additional file 12a), which resulted from receiving more aggressive treatment because of the high percentage of internal organs involved. Conversely, cluster 3 was characterized by lower levels of serum inflammation and immunoglobulin compared with cluster 1, and a higher percentage of superficial organs involved in contrast to cluster 2 (Fig. 3).

In addition, the sex ratios were different between cluster 2 and cluster 3. We have known that IgG4-RD has a gender predilection [1]. Female patients present more frequently with superficial organ involvement, while male patients more frequently have internal organ involvement [20]. Similarly, our cluster analysis results indicated that cluster 2 was characterized by internal organ-dominant (male to female, 3.53:1) and cluster 3 was characterized by superficial organ-dominant (male to female, 0.94:1). Although there were many common characteristics in the male and female clusters after reanalysis in different genders (Additional files 5, 6, and 7), we found some subtle but interesting differences. The relationship between the organs involved and inflammation or disease activity in male patients were closer to those in the total number of patients, while in female patients showed several heterogenicities. And IgG4-RD CS had superior ability to guide treatment in female patients (Additional file 8). The RI score for an organ site at baseline is 3 [5], no matter it is internal organ or superficial organ except for a particular or organ/site which is considered urgent. Thus, female patients characterized by superficial organs-dominant will receive overestimated RI scores compared with male patients.

As for treatment of total patients, patients in cluster 3 had the highest probability to receive “watchful waiting” (Fig. 4a). At the same time, cluster 3 had a shorter half-relapse time of about 24 months (Additional file 12a), which probably attributes to not only lighter treatment, but also lower disease attentions from patients. Therefore, close clinical follow-up and/or regular imaging checks are highlighted in IgG4-RD “watchful waiting” [38, 39]. No difference in RI among clusters 1–3 indicates that baseline heterogeneity exists in patients with a similar level of RI.

IgG-RD CS as a comprehensive score has several unique advantages. Firstly, it is more comprehensive. It was calculated from 22 baseline variables representing the disease heterogeneity presumably driven by differences in underlying molecular pathology better than RI. Secondly, IgG4-RD CS identified clinical subgroups perfectly (Additional file 4 b-c) and was comparable to RI in assessing disease severity, reflecting and corresponding with treatment plans, and predicting drug responses and disease relapse (Fig. 4, Additional files 9, 10, 11, and 12), which also confirmed the value of IgG4-RD CS as a practical comprehensive assessment. Thirdly, IgG4-RD CS was potentially a novel disease assessment tool in clinical practice, because it is computable and objective. The application of RI may be difficult if there are serum-negative patients, or the organ site score is subjective and even not very accurate especially without whole body organ screening. IgG4-RD CS enables assessment and clinical predictions to be made quickly, without the requirement for sophisticated analyses. And the independents introduced in IgG4-RD CS prediction model are supported by previous evidences. Such abnormalities of specific lymphocyte subsets are present in the blood of patients with IgG4-RD, including CD4+ cytotoxic T lymphocytes (CTL), follicular helper T (Tfh) cell, CD19+CD24−CD38hi plasmablasts/plasma cells, and so on [32, 40–44], and the role of Eosinophil in IgG4-RD has been reported in previous studies [15, 16, 45, 46]. Finally, after we performed cluster analysis in different genders, IgG4-RD CS presented superior ability to guide treatment in female patients.

Our study has several limitations. First, it was exploratory and the “IgG4-RD CS” prediction model was not validated by investigation of other cohorts. Second, we did not find significant differences in remission induction or disease relapse among groups (Additional file 11). It may be caused by individual treatment, as data collected from a real world study are different in terms of the follow-up time and other confounding factors. Finally, some variables with a potential role in the pathogenesis of IgG4-RD were not investigated, such as ANA, RF, and neutrophils [26, 47–51].

## Conclusions

In conclusion, according to our definition, the baseline phenotypes of IgG4-RD consisted of inflammation-related axis, disease severity-related axis, and allergy-related axis abnormalities and 154 newly diagnosed patients with IgG4-RD could be classified into three clusters: cluster 1, inflammation and immunoglobulin-dominant group; cluster 2, internal organ-dominant group; and cluster 3, inflammation and immunoglobulin-low with superficial organ-dominant group. In addition, there were significant differences in serum and clinical characteristics among subgroups based on IgG4-RD CS, which indicated that the IgG4-RD CS had the ability to assess disease severity. Subsequently, the “IgG4-RD CS” prediction model introduced 4 independent variables and needs further validation. Accumulation of further evidence along these lines will not only contribute to the identification of clinical phenotypes, but also help in the elucidation of the pathogenesis of IgG4-RD.

## Supplementary information

**Additional file 1.** Baseline characteristics of patients with IgG4-RD (*n* = 154).

**Additional file 2.** Eigenvalue and variance contribution.

**Additional file 3.** Correlation coefficient of each phenotype after extracting 3 PCs.

**Additional file 4.** Other differences among Clusters. a, Distribution of the grades of complement in clusters. Normal values of C3 and C4 were 0.73-1.46 and 0.1-0.4(g/L), respectively. b, Distribution of the grades of IgG4-RD CS in clusters. (CS1, range-1.5 to -0.3; CS2, range -0.3 to 0.9; CS3, range 0.9 to 2.1). c, IgG4-RD CS was compared between clusters. *, *P* value <0.05; ***, P value <0.001.

**Additional file 5.** Three clusters of patients identified by cluster analysis in male and female IgG4-RD patients. cluster1, inflammation and immunoglobulin-dominant group; cluster2, internal organs-dominant group; cluster3, superficial organs-dominant group.

**Additional file 6.** Comparisons of baseline characteristics among three clusters of male IgG4-RD patients. *, P value <0.05; **, P value <0.01; ***, P value <0.001.

**Additional file 7.** Comparisons of baseline characteristics among three clusters of female IgG4-RD patients. Inter.ratio, proportion of internal organs to total organs involved. *, P value <0.05; **, P value <0.01; ***, P value <0.001.

**Additional file 8.** Disease severity scores (mean ± SD) and percentages of different treatments in male and female IgG-RD patients. a, IgG4-RD CS and IgG4-RD RI in male patients; b, IgG4-RD CS and IgG4-RD RI (mean±SD) in female patients; c, Percentages of different treatments in male patients; d, Percentages of different treatments in female patients.

**Additional file 9.** Comparisons of baseline characteristics among CS1-3 grouped by IgG4-RD composite score. *, P value <0.05; **, P value <0.01; ***, P value <0.001.

**Additional file 10.** Baseline characteristics of patients with IgG4-RD grouped by IgG4-RD CS.

**Additional file 11.** Comparisons of remission induction and disease relapse among subgroups. a-b, the remission induction and disease relapse among different subgroups were shown in pie charts.

**Additional file 12.** Comparisons of relapse rate among subgroups. a-c, No difference in cumulative relapse rate by Kaplan-Meier curves. d-i, Real-time ratio of non-relapse patients in follow-up patients. The horizontal axis showed follow-up time (month), the left vertical axis showed the number of patients, the right vertical axis showed the real-time ratio of non-relapse patients in follow-up patients. Black dot, the real-time number of patients in following up; Red dot, the real-time number of relapse patients in following up. The blue curve showed the dynamic changes of the real-time ratio of non-relapse patients in follow-up patients.

**Additional file 13.** Residuals plot of the IgG4-RD CS prediction model by multiple linear regression. a, Residuals were shown with histogram; b, Residuals appeared completely random showed homoscedasticity.

## Data Availability

The datasets used and/or analyzed during the current study are available from the corresponding author on reasonable request.

## Abbreviations

C3: Complement 3
C4: Complement 4
CR: Complete response
CS: Composite score
CTL: Cytotoxic T lymphocytes
ESR: Erythrocyte sedimentation rate
GCs: Glucocorticoids
Hb: Hemoglobin
hsCRP: Hyper-sensitivity C-reactive protein
Ig: Immunoglobulin
IgG4-RD: IgG4-related disease
IgG4-RKD: IgG4-related kidney disease
IMs: Immunosuppressive agents
NC: No change
PCA: Principal components analysis
PCs: Principal components
Plt: Platelet count
PR: Partial response
RI: IgG4-RD responder index
RTX: Rituximab
Tfh: Follicular helper T cell
