# Supplementary material for: Identifying clinical subgroups in IgG4-related disease patients using cluster analysis and IgG4-RD composite score
Source: Arthritis Res Ther. 2020 Jan 10;22:7. doi: 10.1186/s13075-019-2090-9 (PMC6954570; doi:10.1186/s13075-019-2090-9)
Supplement: Supplementary file 1 — Additional file 1. Baseline characteristics of patients with IgG4-RD (n = 154). [file 13075_2019_2090_MOESM1_ESM.docx]

**Additional file 1** Baseline characteristics of patients with IgG4-RD (n = 154)

| **Variable** | **Value** |
| --- | --- |
| Sex (male:female) | 1.8:1 |
| Age (years) | 53.35±13.17 |
| Disease duration (months) | 11.5 (3.75, 36） |
| IgG4-RD RI | 11.5 (7, 15) |
| Allergy history, n (%) | 68 (44.16%) |
| Number of total organs involved, n (%) |  |
| 1 ~ 2 | 65 (42.21%) |
| 3 ~ 4 | 58 (37.66%) |
| ≥ 5 | 31 (20.13%) |
| Number of Superficial organs involved, n (%) |  |
| 0 | 48 (31.17%) |
| 1 ~ 2 | 67 (43.51%) |
| 3 ~ 4 | 39 (25.32%) |
| Number of Internal organs involved, n (%) |  |
| 0 | 46 (29.87%) |
| 1 ~ 2 | 89 (57.79%) |
| 3 ~ 4 | 19 (12.34%) |
| Laboratory tests at baseline |  |
| WBC (x10^9^/L) | 6.53 (5.68,7.89) |
| Eosinophils (x10^9/L) | 0.23 (0.11, 0.42) |
| Eosinophils(%) | 3.35 (1.78,6.13) |
| Lymphocyte (x10^9/L) | 1.92 (1.57,2.42) |
| Lymphocyte(%) | (30.35±9.73) |
| Hemoglobin(g/L) | 135 (125,149.25) |
| Plt (x10^9/L) | 241.5 (201,284) |
| ESR (mm/h) | 18 (7, 51) |
| CRP (mg/L) | 2.05 (0.7,8.38) |
| IgG (g/L) | 19.07 (14.85,23.47) |
| IgA (g/L) | 2.11 (1.44,2.75) |
| IgM (g/L) | 0.89 (0.57,1.30) |
| IgG1 (mg/L) | 9275 (7710,11550) |
| IgG2 (mg/L) | 6060 (4895,7905) |
| IgG3 (mg/L) | 432 (228.5,775.25) |
| IgG4 (mg/L) | 8770 (3557.5,14725) |
| IgE (kU/L) | 342 (145,773) |
| C3 (g/L) | 0.967 (0.78, 1.16) |
| Number of patients with low C3 | 31 (20.13%) |
| Number of patients with normal C3 | 112 (72.73%) |
| Number of patients with high C3 | 11 (7.14%) |
| C4 (g/L) | 0.18 (0.12,0.24) |
| Number of patients with low C4 | 30 (19.5%) |
| Number of patients with normal C4 | 120 (77.9%) |
| Number of patients with high C4 | 4 (2.6%) |

The continuous normally distributed data are presented as mean±standard deviation, non-normally distributed data are presented as median (first quartile, third quartile).
