# Supplementary material for: Identifying clinical subgroups in IgG4-related disease patients using cluster analysis and IgG4-RD composite score
Source: Arthritis Res Ther. 2020 Jan 10;22:7. doi: 10.1186/s13075-019-2090-9 (PMC6954570; doi:10.1186/s13075-019-2090-9)
Supplement: Supplementary file 2 — Additional file 2. Eigenvalue and variance contribution. [file 13075_2019_2090_MOESM2_ESM.docx]

**Additional file 2** Eigenvalue and variance contribution

| **Total Variance Explained** | | | | | |
| --- | --- | --- | --- | --- | --- |
| component | Initial Eigenvalues | | | | |
|  | eigenvalue | | contribution % | | Cumulative % |
| 1 | 4.317 | 19.623 | | 19.623 | |
| 2 | 3.794 | 17.246 | | 36.869 | |
| 3 | 2.330 | 10.590 | | 47.458 | |
| 4 | 1.798 | 8.173 | | 55.632 | |
| 5 | 1.468 | 6.675 | | 62.306 | |
| 6 | 1.230 | 5.590 | | 67.897 | |
| 7 | 1.198 | 5.448 | | **73.344** | |
| 8 | 0.915 | 4.158 | | 77.502 | |
| 9 | 0.841 | 3.824 | | 81.326 | |
| 10 | 0.713 | 3.242 | | 84.568 | |
| 11 | 0.617 | 2.804 | | 87.372 | |
| 12 | 0.572 | 2.598 | | 89.970 | |
| 13 | 0.530 | 2.408 | | 92.377 | |
| 14 | 0.418 | 1.900 | | 94.277 | |
| 15 | 0.345 | 1.568 | | 95.845 | |
| 16 | 0.272 | 1.236 | | 97.081 | |
| 17 | 0.241 | 1.097 | | 98.178 | |
| 18 | 0.159 | 0.724 | | 98.903 | |
| 19 | 0.131 | 0.597 | | 99.500 | |
| 20 | 0.049 | 0.223 | | 99.722 | |
| 21 | 0.039 | 0.175 | | 99.898 | |
| 22 | 0.023 | 0.102 | | 100.000 | |
