# Supplementary material for: Identifying clinical subgroups in IgG4-related disease patients using cluster analysis and IgG4-RD composite score
Source: Arthritis Res Ther. 2020 Jan 10;22:7. doi: 10.1186/s13075-019-2090-9 (PMC6954570; doi:10.1186/s13075-019-2090-9)
Supplement: Supplementary file 3 — Additional file 3. Correlation coefficient of each phenotype after extracting 3 PCs. [file 13075_2019_2090_MOESM3_ESM.docx]

**Additional file 3** Correlation coefficient of

each phenotype after extracting 3 PCs

| **Component Matrix^a^** | | | |
| --- | --- | --- | --- |
|  | component | | |
|  | 1 | 2 | 3 |
| ESR | 0.883 | 0.096 | -0.142 |
| IgG1 | 0.768 | 0.134 | -0.154 |
| CRP | 0.714 | -0.406 | -0.114 |
| IgG | 0.708 | 0.513 | -0.120 |
| Hb | -0.662 | 0.052 | 0.173 |
| Plt | 0.559 | -0.295 | 0.256 |
| IgG3 | 0.532 | 0.455 | -0.192 |
| IgG2 | 0.378 | 0.087 | -0.120 |
| internal.org | 0.248 | 0.192 | -0.087 |
| C3 | 0.152 | -0.765 | 0.205 |
| IgG4 | 0.246 | 0.745 | 0.000 |
| C4 | -0.022 | -0.727 | 0.108 |
| total.org | 0.003 | 0.646 | -0.020 |
| IgA | 0.474 | -0.567 | -0.139 |
| superficial.org | -0.255 | 0.501 | 0.047 |
| IgM | 0.096 | -0.198 | -0.069 |
| Ly.% | -0.050 | 0.118 | -0.048 |
| Eos.% | 0.165 | 0.206 | 0.900 |
| Eos# | 0.115 | 0.328 | 0.818 |
| IgE | 0.236 | 0.154 | 0.533 |
| WBC | 0.398 | -0.303 | 0.408 |
| Ly.# | 0.243 | -0.145 | 0.301 |

^a: Three components were extracted.^
