# Supplementary material for: Identifying clinical subgroups in IgG4-related disease patients using cluster analysis and IgG4-RD composite score
Source: Arthritis Res Ther. 2020 Jan 10;22:7. doi: 10.1186/s13075-019-2090-9 (PMC6954570; doi:10.1186/s13075-019-2090-9)
Supplement: Supplementary file 4 — Additional file 4. Other differences among Clusters. a, Distribution of the grades of complement in clusters. Normal values of C3 and C4 were 0.73-1.46 and 0.1-0.4(g/L), respectively. b, Distribution of the grades of IgG4-RD CS in clusters. (CS1, range-1.5 to -0.3; CS2, range -0.3 to 0.9; CS3, range 0.9 to 2.1). c, IgG4-RD CS was compared between clusters. *, P value <0.05; ***, P value <0.001. [file 13075_2019_2090_MOESM4_ESM.docx]

**Additional file 4** Other differences among Clusters. **a**, Distribution of the grades of complement in clusters. Normal values of C3 and C4 were 0.73-1.46 and 0.1-0.4(g/L), respectively. **b**, Distribution of the grades of IgG4-RD CS in clusters. (CS1, range-1.5 to -0.3; CS2, range -0.3 to 0.9; CS3, range 0.9 to 2.1). **c**, IgG4-RD CS was compared between clusters. *, P value <0.05; ***, P value <0.001.
