# Supplementary material for: Identifying clinical subgroups in IgG4-related disease patients using cluster analysis and IgG4-RD composite score
Source: Arthritis Res Ther. 2020 Jan 10;22:7. doi: 10.1186/s13075-019-2090-9 (PMC6954570; doi:10.1186/s13075-019-2090-9)
Supplement: Supplementary file 5 — Additional file 5. Three clusters of patients identified by cluster analysis in male and female IgG4-RD patients. cluster1, inflammation and immunoglobulin-dominant group; cluster2, internal organs-dominant group; cluster3, superficial organs-dominant group. [file 13075_2019_2090_MOESM5_ESM.docx]

**Additional file 5** Three clusters of patients identified by cluster analysis in male and female IgG4-RD patients. cluster1, inflammation and immunoglobulin-dominant group; cluster2, internal organs-dominant group; cluster3, superficial organs-dominant group.
