# Supplementary material for: Identifying clinical subgroups in IgG4-related disease patients using cluster analysis and IgG4-RD composite score
Source: Arthritis Res Ther. 2020 Jan 10;22:7. doi: 10.1186/s13075-019-2090-9 (PMC6954570; doi:10.1186/s13075-019-2090-9)
Supplement: Supplementary file 6 — Additional file 6. Comparisons of baseline characteristics among three clusters of male IgG4-RD patients. *, P value <0.05; **, P value <0.01; ***, P value <0.001. [file 13075_2019_2090_MOESM6_ESM.docx]

**Additional file 6** Comparisons of baseline characteristics among three clusters of male IgG4-RD patients. *, P value <0.05; **, P value <0.01; ***, P value <0.001.
