# Supplementary material for: Identifying clinical subgroups in IgG4-related disease patients using cluster analysis and IgG4-RD composite score
Source: Arthritis Res Ther. 2020 Jan 10;22:7. doi: 10.1186/s13075-019-2090-9 (PMC6954570; doi:10.1186/s13075-019-2090-9)
Supplement: Supplementary file 8 — Additional file 8. Disease severity scores (mean ± SD) and percentages of different treatments in male and female IgG-RD patients. a, IgG4-RD CS and IgG4-RD RI in male patients; b, IgG4-RD CS and IgG4-RD RI (mean±SD) in female patients; c, Percentages of different treatments in male patients; d, Percentages of different treatments in female patients. [file 13075_2019_2090_MOESM8_ESM.docx]

**Additional file 8** Disease severity scores (mean±SD) and percentages of different treatments in male and female IgG-RD patients. **a**, IgG4-RD CS and IgG4-RD RI in male patients; **b**, IgG4-RD CS and IgG4-RD RI (mean±SD) in female patients; **c**, Percentages of different treatments in male patients; **d**, Percentages of different treatments in female patients.
