# Supplementary material for: Identifying clinical subgroups in IgG4-related disease patients using cluster analysis and IgG4-RD composite score
Source: Arthritis Res Ther. 2020 Jan 10;22:7. doi: 10.1186/s13075-019-2090-9 (PMC6954570; doi:10.1186/s13075-019-2090-9)
Supplement: Supplementary file 10 — Additional file 10. Baseline characteristics of patients with IgG4-RD grouped by IgG4-RD CS. [file 13075_2019_2090_MOESM10_ESM.docx]

**Additional file 10** Baseline characteristics of patients with IgG4-RD grouped by IgG4-RD CS

|  | CS1 n=51 | CS2 n=90 | CS3 n=13 | p value |
| --- | --- | --- | --- | --- |
| Sex(male;female) | 2.19:1 | 1.43:1 | 5.5:1 | 0.142 |
| Age(years) | 53.43±12.29 | 53.07±14.05 | 55±10.72 | 0.885 |
| Disease duration(months) | 8 (3,24) | 10 (3.75,36) | 24 (12,55) | 0.449 |
| IgG4-RD RI | 7 (4,10) | 13 (10,17) | 15 (10.5,21.5) | <0.001 |
| Allergy history, n (%) | 19 (37.3%) | 44 (48.9%) | 5 (38.5) | 0.373 |
| Number of total organs involved | 2 (1,3) | 4 (2,5) | 3 (2.5,6.5) | <0.001 |
| Number of internal organs | 1 (0,1) | 2 (0,2) | 2 (1,2.5) | <0.001 |
| Number of superficial organs | 1 (0,2) | 2 (0,3) | 1 (1,3) | 0.014 |
| Internal organs ratio | 0.5 (0,1) | 0.4 (0,0.67) | 0.4 (0.33,0.5) | 0.929 |
| Laboratory test at baseline |  |  |  |  |
| WBC (x10^9/L) | 6.13 (5.1,7.22) | 6.94 (5.84,8.19) | 7.6 (5.39,8.81) | 0.033 |
| Eosinophils (x10^9/L) | 0.17 (0.07,0.25) | 0.26 (0.15,0.5) | 0.69 (0.26,0.9) | <0.001 |
| Eosinophils (%) | 2.5 (1.2,4.2) | 3.75 (1.90,6.83) | 5.5 (3.45,12.1) | 0.001 |
| Lymphocyte (x10^9/L) | 1.62 (1.39,1.94) | 2.09 (1.61,2.6) | 2 (1.95,2.39) | 0.001 |
| Lymphocyte (%) | 28.9±10.79 | 31.15±9.21 | 30.47±8.84 | 0.42 |
| Hemoglobin(g/L) | 145 (132,153) | 133.5 (121,147.5) | 125 (108,129.5) | <0.001 |
| Plt (x10^9/L) | 215 (171,254) | 250 (209.75,301.75) | 276 (217,337.5) | <0.001 |
| ESR (mm/h) | 8 (5,18) | 25.5 (10.75,57.25) | 82 (72,112.5) | <0.001 |
| CRP (mg/L) | 1.28 (0.34,6.3) | 2.08 (0.78,9.28) | 9.21 (2.21,46.28) | 0.005 |
| IgG (g/L) | 14.93 (13.27,18.4) | 20.13 (16.54,23.92) | 45.9 (34.84,54.12) | <0.001 |
| IgA (g/L) | 2.28 (1.57,2.74) | 2.09 (1.45,2.77) | 1.09 (0.69,3.78) | 0.216 |
| IgM (g/L) | 1.04 (0.64,1.37) | 0.82 (0.55,1.25) | 0.58 (0.31,2.13) | 0.432 |
| IgG1 (mg/L) | 8190 (6820,9710) | 9380 (7800,11425) | 17600 (13300,31550) | <0.001 |
| IgG2 (mg/L) | 5540 (4480,7130) | 6205 (5100,8092.5) | 7680 (4405,11650) | 0.101 |
| IgG3 (mg/L) | 301 (180,558) | 495 (230.5,792) | 1440 (1000,2780) | <0.001 |
| IgG4 (mg/L) | 3770 (2070,6910) | 11800 (5412.5,17075) | 24800 (8465,56500) | <0.001 |
| IgE (kU/L) | 220 (59.8,442) | 436.5 (208,1010.5) | 712 (235,1774) | <0.001 |
| C3 (g/L) | 1.018 (0.86,1.15) | 0.96 (0.77,1.17) | 0.46 (0.33,1.16) | 0.015 |
| C4 (g/L) | 0.2 (0.16,0.26) | 0.17 (0.1,0.24) | 0.05 (0.02,0.17) | <0.001 |

IgG4-RD CS grades (CS1, range-1.5 to -0.3; CS2, range -0.3 to 0.9; CS3, range 0.9 to 2.1); CS1-3, the groups in which patients with IgG4-RD CS grades of grade1,2,3 accordingly.
