# Supplementary material for: Identifying clinical subgroups in IgG4-related disease patients using cluster analysis and IgG4-RD composite score
Source: Arthritis Res Ther. 2020 Jan 10;22:7. doi: 10.1186/s13075-019-2090-9 (PMC6954570; doi:10.1186/s13075-019-2090-9)
Supplement: Supplementary file 11 — Additional file 11. Comparisons of remission induction and disease relapse among subgroups. a-b, the remission induction and disease relapse among different subgroups were shown in pie charts. [file 13075_2019_2090_MOESM11_ESM.docx]

**Additional file 11** Comparisons of remission induction and disease relapse among subgroups. **a-b**, the remission induction and disease relapse among different subgroups were shown in pie charts.
