# Supplementary material for: Identifying clinical subgroups in IgG4-related disease patients using cluster analysis and IgG4-RD composite score
Source: Arthritis Res Ther. 2020 Jan 10;22:7. doi: 10.1186/s13075-019-2090-9 (PMC6954570; doi:10.1186/s13075-019-2090-9)
Supplement: Supplementary file 12 — Additional file 12. Comparisons of relapse rate among subgroups. a-c, No difference in cumulative relapse rate by Kaplan-Meier curves. d-i, Real-time ratio of non-relapse patients in follow-up patients. The horizontal axis showed follow-up time (month), the left vertical axis showed the number of patients, the right vertical axis showed the real-time ratio of non-relapse patients in follow-up patients. Black dot, the real-time number of patients in following up; Red dot, the real-time number of relapse patients in following up. The blue curve showed the dynamic changes of the real-time ratio of non-relapse patients in follow-up patients. [file 13075_2019_2090_MOESM12_ESM.docx]

**Additional file 12** Comparisons of relapse rate among subgroups. **a-c**, No difference in cumulative relapse rate by Kaplan-Meier curves. **d-i**, Real-time ratio of non-relapse patients in follow-up patients. The horizontal axis showed follow-up time (month), the left vertical axis showed the number of patients, the right vertical axis showed the real-time ratio of non-relapse patients in follow-up patients. Black dot, the real-time number of patients in following up; Red dot, the real-time number of relapse patients in following up. The blue curve showed the dynamic changes of the real-time ratio of non-relapse patients in follow-up patients.
