# Supplementary material for: Identifying clinical subgroups in IgG4-related disease patients using cluster analysis and IgG4-RD composite score
Source: Arthritis Res Ther. 2020 Jan 10;22:7. doi: 10.1186/s13075-019-2090-9 (PMC6954570; doi:10.1186/s13075-019-2090-9)
Supplement: Supplementary file 13 — Additional file 13. Residuals plot of the IgG4-RD CS prediction model by multiple linear regression. a, Residuals were shown with histogram; b, Residuals appeared completely random showed homoscedasticity. [file 13075_2019_2090_MOESM13_ESM.docx]

**Additional file 13** Residuals plot of the IgG4-RD CS prediction model by multiple linear regression. **a**, Residuals were shown with histogram; **b**, Residuals appeared completely random showed homoscedasticity.
